# Innovative and Highly Sensitive Detection of *Clostridium perfringens* Enterotoxin Based on Receptor Interaction and Monoclonal Antibodies

**DOI:** 10.3390/toxins13040266

**Published:** 2021-04-08

**Authors:** Thea Neumann, Maren Krüger, Jasmin Weisemann, Stefan Mahrhold, Daniel Stern, Martin B. Dorner, Cécile Feraudet-Tarisse, Christopher Pöhlmann, Katharina Schulz, Ute Messelhäußer, Dagmar Rimek, Frank Gessler, Thomas Elßner, Stéphanie Simon, Andreas Rummel, Brigitte G. Dorner

**Affiliations:** 1 Biological Toxins, Centre for Biological Threats and Special Pathogens, Robert Koch Institute (RKI), Seestr. 10, 13353 Berlin, Germany; Thea.Neumann@gmx.de (T.N.); KruegerM@rki.de (M.K.); SternD@rki.de (D.S.); DornerM@rki.de (M.B.D.); 2 Institut für Toxikologie, Medizinische Hochschule Hannover (MHH), Carl-Neuberg-Str. 1, 30625 Hannover, Germany; Weisemann.Jasmin@mh-hannover.de (J.W.); Mahrhold.Stefan@mh-hannover.de (S.M.); rummel.andreas@mh-hannover.de (A.R.); 3 Medicines and Healthcare Technologies Department (DMTS), Paris-Saclay University, French Alternative Energies and Atomic Energy Commission (CEA), INRAE, SPI, 91191 Gif-sur-Yvette, France; cecile.feraudet-tarisse@cea.fr (C.F.-T.); Stephanie.simon@cea.fr (S.S.); 4 Bruker Daltonik GmbH, Permoserstr. 15, 04318 Leipzig, Germany; poehlmann@senslab.de (C.P.); katharina.ks@gmx.de (K.S.); Thomas.Elssner@bruker.com (T.E.); 5 Bavarian Health and Food Safety Authority, Veterinärstr. 2, 85764 Oberschleißheim, Germany; Ute.Messelhaeusser@lgl.bayern.de; 6 Thuringian State Authority for Consumer Protection, Tennstedter Straße 8/9, 99947 Bad Langensalza, Germany; Dagmar.Rimek@tlv.thueringen.de; 7 miprolab GmbH, Marie-Curie-Str. 8, 37079 Göttingen, Germany; gessler@miprolab.com

**Keywords:** *C. perfringens* enterotoxin CPE, *Clostridium perfringens*, monoclonal antibodies, receptor claudin-4, stationary lab-based detection, mobile on-site detection

## Abstract

*Clostridium perfringens* enterotoxin (CPE) regularly causes food poisoning and antibiotic-associated diarrhea; therefore, reliable toxin detection is crucial. To this aim, we explored stationary and mobile strategies to detect CPE either exclusively by monoclonal antibodies (mAbs) or, alternatively, by toxin-enrichment via the cellular receptor of CPE, claudin-4, and mAb detection. Among the newly generated mAbs, we identified nine CPE-specific mAbs targeting five distinct epitopes, among them mAbs recognizing CPE bound to claudin-4 or neutralizing CPE activity in vitro. In surface plasmon resonance experiments, all mAbs and claudin-4 revealed excellent affinities towards CPE, ranging from 0.05 to 2.3 nM. Integrated into sandwich enzyme-linked immunosorbent assays (ELISAs), the most sensitive mAb/mAb and claudin-4/mAb combinations achieved similar detection limits of 0.3 pg/mL and 1.0 pg/mL, respectively, specifically detecting recombinant CPE from spiked feces and native CPE from 30 different *C. perfringens* culture supernatants. The implementation of mAb- and receptor-based ELISAs into a mobile detection platform enabled the fast detection of CPE, which will be helpful in clinical laboratories to diagnose diarrhea of assumed bacterial origin. In conclusion, we successfully employed an endogenous receptor and novel high affinity mAbs for highly sensitive and specific CPE-detection. These tools will be useful for both basic and applied research.

## 1. Introduction

The Gram-positive anaerobe bacterium *Clostridium perfringens* can cause severe diseases in humans and animals including gas gangrene, necrotic enteritis, food poisoning and non-foodborne gastrointestinal illnesses [1,2]. Mainly due to its ability to form resistant spores, *C. perfringens* is ever-present in nature and survives in many environmental niches such as soil, sewage, foods, and the intestines of humans and animals [3,4,5]. Essentially, the virulence of this bacterium arises from the broad spectrum of more than 20 toxins and exoenzymes produced [3,6]. Implicating characteristic diseases, the toxins α, β, ε, ι, *Clostridium perfringens* enterotoxin (CPE) and necrotic enteritis toxin (NetB) provide the basis for a typing scheme dividing *C. perfringens* isolates into seven distinct toxinotypes A–G (the former typing scheme contained five strains A–E [7]). The toxins from the typing scheme exert divergent modes of action, relevant are in particular the phospholipase activity of α-toxin [8], ADP-ribosylation by the binary ι-toxin [9,10] as well as pore formation by β-toxin [11], NetB [12], ε-toxin [13], and CPE [14], highlighting the enormous range and variability of toxins produced by *C. perfringens*.

Among the seven toxinotypes, *Clostridium perfringens* enterotoxin (CPE) produced by type C, D, E, and F strains [15,16] is of considerable clinical importance. As the causative agent of *C. perfringens* type F food poisoning [17], CPE is responsible for approximately 1 million cases of foodborne illnesses per year in the United States [18]. CPE can serve as a diagnostic marker in the stools of patients to demonstrate the involvement of *C. perfringens* in food poisoning [19]. Furthermore, enterotoxigenic *C. perfringens* type F strains producing CPE mediate several non-foodborne gastrointestinal diseases including many cases of antibiotic-associated diarrhea (AAD) [20,21], sporadic diarrhea (SD), and nosocomial diarrheal disease [22,23].

The key symptoms of CPE-associated diseases comprising intestinal cramps and watery diarrhea without fever or vomiting [24,25] result from the toxin’s pore forming activity. Consisting of a C-terminal receptor binding domain (RBD) and an N-terminal pore-forming domain (PFD) involved in oligomerization and pore formation [14,26], CPE belongs to the aerolysin family of pore-forming toxins [27]. The interaction of CPE with endogenous receptors of the claudin family initiates the toxin’s oligomerization, its assembly into distinct complexes consisting of CPE, receptor, and non-receptor proteins and ultimately the formation of a membrane pore [28,29,30]. These cellular actions alter the permeability of the plasma membrane [31,32], induce calcium influx and lead to CPE-induced cell death [33,34]. A crucial step in the mode of action is the binding of CPE to the cellular protein family of claudins. As cell–cell adhesion molecules, these four-transmembrane domain proteins essentially shape the tight junctions and contribute to the epithelium’s barrier function [35]. Among the 27 human claudin isoforms described [36], CPE interacts strongly with claudin-4 and to a lesser extent with claudin-3 and murine claudin-19 [37,38,39]. Additionally, claudin-6, -7, -8, -9, and -14 are reported to be low affinity receptors for CPE [40].

For the diagnosis of gastrointestinal diseases with suspected involvement of CPE, the reference method EN ISO 7937 for the enumeration of presumptive *C. perfringens* colonies is performed routinely from food and feed and comprises the cultivation in specific media and enumeration of characteristic colonies [41]. Other methods identify *C. perfringens* isolates on the basis of characteristic peptide fingerprints by mass spectrometry [42,43]. While these approaches indicate the mere presence of *C. perfringens* in food samples or feces of patients, PCR-based toxinotyping tests [7,44] or *cpe* gene detection assays [45,46] prove the presence of toxin genes and therefore the potential clinical significance of the isolated strains. However, detection of the *cpe* gene fails to provide unambiguous evidence for the actual production of CPE by the *C. perfringens* strain under analysis. On the one hand, there are silent *cpe* genes described not encoding for a complete CPE, and on the other hand, CPE expression only occurs during sporulation of *C. perfringens* and requires external trigger factors [47]. Thus, assays aiming at toxin detection would allow for a more reliable assessment of a strain’s pathogenic potential. Unlike many other clostridial toxins, there is little to no evidence for a variability in CPE’s amino acid sequence [15]. Therefore, immunological methods employing highly specific monoclonal antibodies can be applied to detect CPE. In this respect, few enzyme-linked immunosorbent assays (ELISAs) as well as rapid and mobile detection assays such as lateral flow assays (LFAs) have been described, including commercial test systems [21,48,49,50,51]. Alongside well-established LFAs, more recently developed fast on-site detection platforms such as the “portable BioDetector integrated” (pBDi, Bruker Optik GmbH, Leipzig, Germany) used in this work offer additional benefits: this fully automated device enables the multiplex detection of several analytes simultaneously via electrochemical biochips carrying immobilized capture reagents on its 16 interdigitated gold electrodes [52]. Using antibodies as capture reagents, measurements can be performed in a sandwich ELISA format. Instead of the optically readable color change used in conventional, stationary ELISA performed in microtiter plates, the pBDi system detects a current derived from the oxidation of an electro-active product generated by enzymatic conversion of an electro-inactive substrate. A redox recycling process between interdigitated gold electrodes recycles electro-active product and results in signal amplification.

The objective of this work was (i) to employ an optimized endogenous receptor claudin-4, a member of the tetraspanin superfamily, for the functional detection of CPE, (ii) to compare the performance of receptor-based and conventional antibody-based tools and detection systems for CPE, and (iii) to transfer conventional ELISA approaches to the automated mobile detection platform pBDi. To this end, we developed conventional antibody-based sandwich ELISAs comprising two monoclonal antibodies, and receptor-based sandwich ELISAs deploying optimized claudin-4 as the capture reagent and a detection antibody. Both the antibody-based and the receptor-based approach achieved highly sensitive detection of CPE, revealing detection limits of 0.3 pg/mL and 1.0 pg/mL, respectively. Furthermore, we demonstrated that both assay formats specifically detected native CPE derived from all 30 *cpe*-positive strains out of a total of 64 *C. perfringens* supernatants in a blinded test. The implementation of the detection assays into the pBDi system enabled fast on-site detection of CPE with detection limits in the ng/mL-range. Both the antibody-based and the receptor-based assays were clearly able to detect recombinant CPE from spiked stool samples in in the stationary and the on-site detection platforms.

## 2. Results

### 2.1. Generation of High Affinity Monoclonal Antibodies (mAbs) Recognizing Distinct Epitopes 

The aim of this work was to explore and compare antibody- and receptor-based immunoassays for the sensitive detection of CPE. On the one hand, a set of mAbs was selected to establish classical sandwich ELISA (comprising capture and detection mAbs). Apart from that, for a receptor-based ELISA using recombinant claudin-4 (based on construct GST-ShCLDN4H8, see Material and Methods) as the capture reagent, corresponding detection mAbs were selected which recognize a distant epitope not utilized by the receptor. To this end, we immunized mice with recombinant CPE D48A mutant with reduced toxicity [53] or recombinant CPE RBD, monitored their antibody titers and applied hybridoma technology to produce mAbs. Out of the 2211 hybridomas screened, we specifically selected seven mAbs employing ELISA (indirect and sandwich format) and surface plasmon resonance (SPR) to obtain information on binding kinetics and epitope groups. In an alternative immunization approach, two further CPE-specific antibodies were selected after immunization with formalin-detoxified recombinant CPE.

Altogether, we generated a panel of nine antibodies of subclass IgG1 recognizing five distinct CPE epitopes (Table 1). SPR-based epitope binning was applied where one specific antibody was captured on a CM5 chip via anti-mouse IgG antibodies (Supplementary Figure S1). After the injection of the antigen and binding to the capture antibody, a second antigen-specific antibody was injected. The second antibody would only be able to bind to the antigen if the corresponding epitope would be accessible, that is, not yet occupied by the first capture antibody. Therefore, a signal increase after the injection of the second antibody only appears when it binds a distinct epitope than the first antibody. This approach revealed that mAbs CPE1, CPE639, CPE562, CPE384, and CPE1339 were directed against distinct epitopes 1, 2, 3, 4, and 5, respectively (arbitrary consecutive numeration of epitopes; for epitope binning curves, see Supplementary Figure S1). The epitope recognition of CPE18 overlapped with epitopes 3 and 4 and the epitope recognition of CPE1339 overlapped with CPE384. CPE9, CPE58, and CPE281 recognized the same epitope 1 as CPE1 and were omitted from Supplementary Figure S1 in favor of clarity of the illustration. As seen in later sandwich ELISA experiments, the receptor claudin-4 occupied the same epitope on CPE as antibodies binding to epitope 1, since it blocked the binding of CPE1 (see Table 2). 

To demonstrate their binding specificity, mAbs were characterized by Western blot analysis using recombinant holo-CPE (aa 26–319, wild type) and the separated CPE domains PFD (aa 26–202, GST-PFD-t-mCherry) and RBD (aa 203–319) as target antigens as well as ε-toxin (Etx) as unrelated antigen (see Supplementary Figure S2). Except for CPE384, all mAbs were applicable in Western blot analysis and specifically bound CPE without cross reactivity against Etx. As shown in Supplementary Figure S2, CPE384 gave no signals on the tested antigens in Western blot analysis and is therefore likely not able to bind denatured CPE. Analysis of the isolated CPE domains enabled us to determine the domain specificity of the mAbs. In this regard, CPE1, CPE9, CPE58, CPE281, and CPE1339 recognized the RBD while CPE639 uniquely recognized the PFD. Since for some mAbs, Western blotting resulted in no (CPE384) or weak detection signals (CPE18, CPE562), further investigation of domain recognition was performed by SPR-based binding measurements using the recombinant RBD and PFD as antigens (for binding curves, see Supplementary Figure S3). SPR analysis confirmed RBD specificity of mAbs CPE1, CPE9, CPE58, CPE281, and CPE1339 and unambiguously identified CPE18, CPE384, CPE562, and CPE639 to be specific for PFD. 

For a more detailed characterization of the mAbs, their ability to neutralize recombinant CPE (aa 26–319, wild type) in vitro was investigated by a cell-based cytotoxicity assay using the xCELLigence system (Supplementary Figure S4). Except for CPE639, all mAbs protected Vero cells exposed to 1 nM CPE (~10 × IC_50_) from cell death at a 100-fold molar excess of mAbs to CPE. At this ratio, all protective antibodies exerted similar inhibitory effects on CPE’s cytotoxicity, reflected by normalized cell indices of 100% or higher. In contrast, incubating Vero cells with CPE pre-incubated with only a 10-fold molar excess of mAbs revealed considerably different inhibitory efficacies of the mAbs. Under these conditions, mAbs CPE58 and CPE281 failed to prevent CPE’s cytotoxicity, but all remaining mAbs protected Vero cells reaching cell indices between 84% (CPE1) and 17% (CPE384). Based on these data, best neutralizing efficacies were observed with CPE1 and CPE9 (Supplementary Figure S4) which block access to the claudin receptor binding site of CPE.

**Figure 1:**
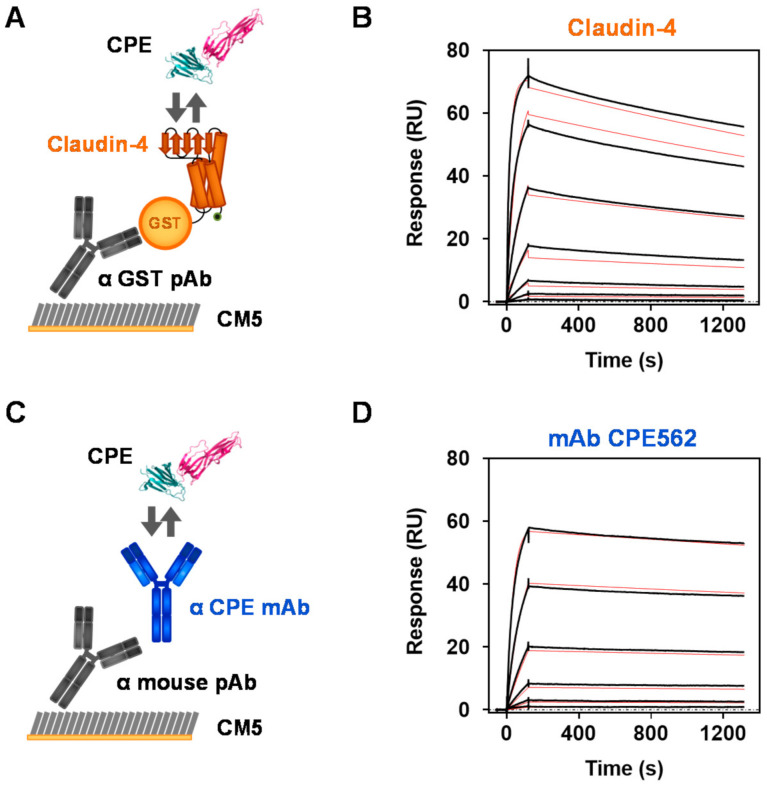
Comparison of binding kinetics between the endogenous receptor claudin-4 or mAb CPE562 to its target CPE. To characterize the interaction between CPE and recombinantly expressed human claudin-4 (**A**,**B**) or a CPE-specific mAb (**C**,**D**), SPR binding experiments were performed. GST-tagged recombinant claudin-4 (GST-ShCLDN4H8) (**A**) was captured on a CM5 chip via an anti-GST polyclonal antibody. Murine mAb CPE562 (**C**) was captured via an anti-mouse polyclonal antibody. Thereafter, serial 1:3 dilutions of recombinant CPE (aa 26–319, wild type; starting with 93 nM) were injected in separately performed runs in a multi-cycle kinetic and measured on a Biacore T-200 instrument. To determine kinetic binding parameters from one representative measurement, the measured binding responses (black lines in **B**,**D**) were fitted (red lines in **B**,**D**) using a 1:1 Langmuir binding model. Kinetic properties summarized in Table 1 were derived from these measurements. CPE structure according to PDB 3AM2. Modified illustration of claudin-4 structure according to Shinoda et al. [46].

Since the sensitivity of the sandwich ELISA crucially depends on the affinity of the mAbs integrated, we determined kinetic binding parameters and affinity constant *K_D_* (calculated from *k_d_*/*k_a_*) by SPR measurements. All mAbs showed high affinity with a *K_D_* ranging from 10^−11^ to 10^−9^ M for recombinant CPE (aa 26–319, wild type; Table 1; for sensorgrams, see Figure 1D and Supplementary Figure S5). While the association rate constants *k_a_* varied in a narrow range (2.7 × 10^5^ M^−1^ s^−1^ ≤ *k_a_* ≤ 1.2 × 10^6^ M^−1^ s^−1^), substantial differences were observed for the dissociation rate constants *k_d_*. For CPE58 and CPE281, fast dissociation of CPE with *k_d_* in 10^−3^ s^−1^ range was measured, indicating unstable binding of the toxin to the antibody. In contrast, the extremely low dissociation rate constants of 5.4 × 10^−5^ s^−1^, 2.6 × 10^−5^ s^−1^, and 1.3 × 10^−5^ s^−1^ for CPE1, CPE9, and CPE1339 resulted in the highest affinities among the mAbs studied, with *K_D_* accounting for 5.3 × 10^−11^, 3.9 × 10^−11^, and 1.1 × 10^−11^ M, respectively (Table 1).

After comprehensive characterization of the CPE-specific mAbs, we set up sandwich ELISAs comprising either two mAbs (antibody-based assay) or explored a new assay format using the recombinant CPE receptor claudin-4 (GST-ShCLDN4H8) for capture in combination with a detection mAb (receptor/mAb-based assay). To this end, one representative mAb was selected from each epitope group and included in further investigations.

### 2.2. Comparing Affinities of the CPE Receptor Claudin-4 with a CPE-Specific Antibody 

An innovative approach of this work was to employ an optimized endogenous receptor protein, the tetraspanin claudin-4, for functional detection of CPE. As demonstrated above, the mAbs display a *K_D_* in the subnanomolar range to their antigen, which is in a suitable range for efficient immunoaffinity enrichment of analytes. A low *K_D_* of 3.4 nM for the interaction of claudin-4 and CPE [54] and the corresponding claudin-4-CPE-RBD co-crystal structure were reported recently [37]. In this publication, the human claudin-4 was expressed in a cell-free system in the presence of lipids so that membrane proteins could assemble into membrane fragments spontaneously, which eventually fused to form liposomes if the detergent concentration was decreased. This approach yielded monodisperse claudin-4 multimers; however, these liposomes might show stability issues over time and may be of limited use with respect to an upscaling in production. Furthermore, a stable direct immobilization of the receptor for the application as a capture reagent in microtiter plates (stationary ELISA) and on gold surfaces (pBDi platform) cannot easily be realized using liposomal structures.

Therefore, we aimed at generating a soluble claudin-4 construct without the need of liposomal stabilization but showing high affinity to its ligand CPE in the low nanomolar range. We recently succeeded in this endeavor and optimized human claudin-4 among a panel of claudin isoforms investigated in parallel as a soluble high affinity receptor candidate comprising necessary tag integrations [55]. This recombinant soluble GST-tagged claudin-4 fusion protein (GST-ShCLND4H8) was expressed and purified for our investigations to precisely determine kinetic binding parameters to recombinant CPE (aa 26–319, wild type) by SPR measurements. For comparison, the SPR binding curves of claudin-4 and of the high affinity mAb CPE562 produced in this work are shown in Figure 1 (for all other mAbs see Supplementary Figure S5; for kinetic binding parameters see Table 1).

Measurements were performed in a multicycle manner: after directed immobilization of claudin-4 via a GST-capture pAb (Figure 1A) or of mAb CPE562 via a mouse-capture pAb (Figure 1B) a 1:3 serial dilution of CPE was injected in separate cycles. Based on the curves obtained (Figure 1B,D), the association (time section 0 to 120 s) and dissociation rate constants (120 s to 1320 s) of the toxin were deduced. While CPE displayed a 1.8-times higher association rate constant to claudin-4 than to CPE562, its dissociation rate constant was three-fold higher for claudin-4 than for CPE562 (for *k_a_* and *k_d_* see Table 1), indicating that the binding of CPE by this recombinant receptor construct is faster but less stable compared to the binding of CPE by mAb CPE562. Calculated from the aforementioned kinetic parameters *k_a_* and *k_d_*, the binding constant *K_D_* for the binding to CPE amounted to 3.8 × 10^−10^ or 2.2 × 10^−10^ M for recombinant claudin-4 or CPE562, respectively (Table 1). In conclusion, recombinant claudin-4 bound CPE with subnanomolar affinity similar to monoclonal antibodies, thereby demonstrating excellent binding kinetics. Due to the observed remarkable affinity, we considered claudin-4 to be a promising capture reagent for a receptor-based sandwich ELISA.

### 2.3. Development of Stationary and Mobile Sandwich ELISA Using High-Affinity mAbs and Receptor Interaction

After a comprehensive characterization of the interactions of CPE with the newly generated mAbs versus the recombinant receptor claudin-4, we combined these reagents to set up stationary sandwich ELISA in a classical 96-well assay format. To this end, either different mAbs or claudin-4 were immobilized onto microtiter plates, incubated with serial dilutions of recombinant CPE (aa 1–319, wild type) and detected with biotinylated mAbs. A colorimetric signal was generated by the subsequent binding of a streptavidin-poly–horseradish peroxidase (HRP) conjugate followed by substrate conversion. Altogether, we tested five claudin-4/mAb and 25 mAb/mAb-combinations (Table 2) applying one selected mAb displaying superior affinity from each of the five epitope groups. To compare these combinations with regard to sensitivity, we evaluated the half maximal effective concentration (EC_50_) values derived from the sigmoidal ELISA dose–response curves obtained. Since CPE is a monomeric molecule in its free soluble form without repetitive epitopes, only antibodies binding distinct epitopes can be combined as capture and detection reagents in a sandwich-based assay format. Accordingly, and in agreement with the data from the epitope binning (Supplementary Figure S1), where no signal increase can be seen if one specific antibody is applied as both capture and detection reagent, only combinations of mAbs recognizing distinct epitopes resulted in the 20 possible mAb/mAb ELISA settings shown in Table 2. A total of 12 of these combinations detected CPE with high sensitivity, as manifested by EC_50_ values lower than 0.2 ng/mL. Likewise, we tested five receptor/mAb-combinations. Combining recombinant claudin-4 as capture reagent with biotinylated mAb CPE562 resulted in a remarkable sensitivity which matched the best mAb/mAb combination CPE1/CPE562 (EC_50_ values < 0.2 ng/mL, both combinations highlighted by bold font in Table 2). In contrast, applying the mAbs CPE1339, CPE384 and CPE639 as detection reagents for the receptor-based assay resulted in significantly lower sensitivities than determined for most of the mAb/mAb combinations (EC_50_ values > 0.5 ng/mL). Among the five antibodies used, only biotinylated CPE1 did not detect claudin-4 captured CPE and was therefore assigned to epitope 1 (see Table 1). As the RBD of the toxin interacts with the receptor claudin-4, we assume that the RBD recognition by the detection antibody is prevented in the CPE-bound claudin-4, leading to the conclusion that epitope 1 corresponds to the claudin-4 receptor binding site in CPE.

**Figure 2:**
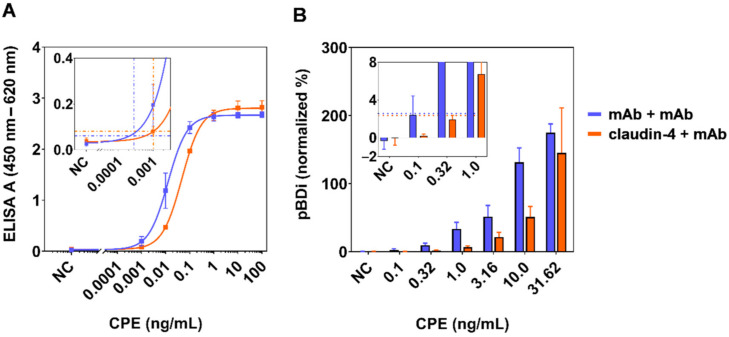
Detection of CPE by classical sandwich ELISA and by the automated on-site detection platform. Combinations of capture reagents CPE1 (blue) or claudin-4 (orange, recombinant construct GST-ShCLDN4H8) and detection with mAb CPE562 (both approaches) were integrated into sandwich ELISA performed on 96-well microtiter plates (**A**) and subsequently transferred into the portable BioDetector integrated (pBDi) system that is based on biochips carrying interdigitated gold electrodes and electrochemical detection (**B**). For (**A**), the capture reagents were immobilized on the surface of microtiter plates, incubated with serial dilutions of recombinant CPE (aa 1–319, wild type) and detected with biotinylated CPE562. Results from five independent experiments are shown (n = 5, mean ± SD). For (**B**), mAb CPE1 and claudin-4 were immobilized in duplicates on the surface of electrochemical biochips. For each concentration of recombinant CPE (aa 1–319, wild type), two biochips with two target positions each were incubated with the antigen and detected by biotinylated CPE562 (n = 4, mean ± SD). The inserts show relevant sections of the graph at low antigen concentrations to illustrate cut-off values (horizontal dashed lines) used to calculate the detection limits (vertical dashed lines).

To compare these combinations and precisely determine the limit of detection (LOD), we repeatedly (n = 5) performed the sandwich ELISA using mAb CPE1 (antibody-based assay) or claudin-4 (receptor-based assay) to capture the toxin and using biotinylated mAb CPE562 for detection (Figure 2A and Table 3). With an LOD of 0.28 pg/mL, the antibody-based ELISA (curves in blue) achieved approximately a four-fold better sensitivity than the receptor-based ELISA (curves in orange) with an LOD of 1.03 pg/mL. In conclusion, the mAb/mAb and the claudin-4/mAb approach exhibited slightly differing but nevertheless excellent sensitivities.

For fast detection within 20 minutes, we integrated the most sensitive antibody- and receptor-based assays into the automated on-site detection platform pBDi [52,56]. To this end, capture reagents diluted in phosphate-buffered saline (PBS) (mAb CPE1) or in PBS supplemented with CHAPS and bovine serum albumin (BSA) (claudin-4, recombinant construct GST-ShCLDN4H8) were immobilized on the surface of biochips and serial 1:3.16 dilutions of recombinant CPE (aa 1–319) were detected by biotinylated mAb CPE562 (Figure 2B). Electrochemical read-out was generated by the subsequent binding of a streptavidin–reporter enzyme conjugate (streptavidin-β-galactosidase) and substrate conversion to a redox-active product. Using the standard deviation from eight blank values, we calculated a detection threshold (dashed lines in insert, Figure 2B). The LOD was defined as the first measured concentration exceeding this threshold. 

As to be seen in Figure 2B, the detection of CPE by the antibody-based approach exhibited an approximately three-fold better sensitivity than the receptor-based approach, which is in good agreement with the sensitivity difference observed in classical ELISA experiments. With LODs of 316 pg/mL and 1000 pg/mL for the antibody-based (blue bars) and the receptor-based assay (orange bars), respectively, the detection limit of the on-site detection system pBDi was approximately three orders of magnitude higher than the classical stationary ELISA but delivered results within 20 min on-site instead of 5 h (Table 3). Furthermore, the assay workflow after sample preparation for pBDi is fully automated, whereas for stationary ELISA several manual assay steps as well as laboratory infrastructure is required. The reduced sensitivity in the automated on-site platform was mainly due to the intrinsic short incubation times and the rapid detection but was still excellent when compared to classical LFAs.

Aside the differences in sensitivity, the on-site platform not only allows for fast detection but also the detection of up to six (in case of double determination and integration of two positive as well as negative control reactions) different antigens in one measurement depending on the design of the biochip. Considering the four-transmembrane domain protein structure of claudin-4, the striking finding in assay integration was that the receptor-based assay format could be transferred from the microtiter plate to the biochip format while reaching a similar performance when compared to the antibody-based format (only three- to four-fold higher LOD, Table 3).

### 2.4. Detection of CPE from Spiked Fecal Samples 

**Figure 3:**
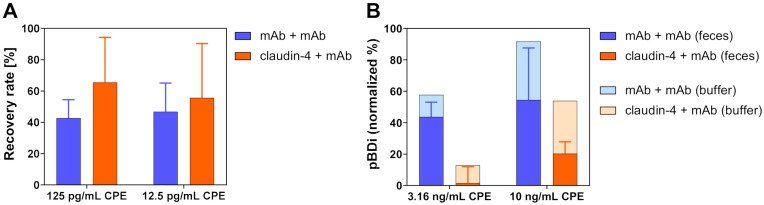
Detection of CPE from spiked fecal samples. Recombinant CPE was spiked into feces extracts and detected by (**A**) ELISA performed in microtiter plates or (**B**) the automated on-site detection platform pBDi using the antibody-based approach (blue, capture reagent mAb CPE1) or the receptor-based approach (orange, capture reagent claudin-4, recombinant construct GST-ShCLDN4H8) and detection with mAb CPE562 (all approaches). For (**A**), 125 pg/mL or 12.5 pg/mL of CPE were separately spiked into feces extracts of three donors and quantified by the microtiter plate-based ELISA. Recovery rates were determined by referencing quantified CPE in feces to quantified CPE in buffer (value in buffer set to 100 %; n = 3, mean ± SD). For (**B**), 3.16 ng/mL or 10 ng/mL of CPE were separately spiked into feces extracts of three donors and measured with the pBDi-based assays. For each concentration of CPE and each feces donor, one biochip with two target positions each was incubated with the samples. For pBDi results, normalized signal intensities for feces samples (dark columns; n = 3, mean ± SD) are shown in comparison to signal intensities in buffer (light columns).

The performance of the newly established assays described in Section 2.3 were determined by measurements of highly purified recombinant CPE in buffer, representing most favorable conditions for toxin detection. In real cases of CPE-induced gastrointestinal disease, fecal samples are important, nevertheless challenging clinical samples for diagnostics. Therefore, the antibody-based and the receptor-based assays, both applied in microtiter plates and in the on-site detection platform pBDi as given in Table 3, were tested for their performance when detecting recombinant CPE (aa 1–319, wild type) spiked into fecal samples of three donors (Figure 3). In the plate-based ELISA, the detection of 12.5 pg/mL (approximately corresponding to the EC_50_ of the mAb/mAb-ELISA) and 125 pg/mL (approximately 10 × EC_50_) resulted in recovery rates of about 40% to 60% of spiked CPE in comparison to the detection of CPE in buffer which was set as 100%. Interestingly, the receptor-based assay seemed to yield slightly higher recovery rates than the mAb/mAb-ELISA. Resulting low background signals were eliminated by measuring fecal extracts without the addition of toxin in parallel in these experiments. Additionally, control experiments applying ELISAs with an irrelevant capture antibody (anti-ricin antibody R109 [57]) or an irrelevant receptor (recombinant receptor synaptotagmin II specific for botulinum neurotoxin B [58]) were applied together with the CPE-detection mAb CPE562 to determine unspecific signals (“control ELISA”). Here, the unspiked feces extracts without toxin gave the same low signals in the specific and in the control ELISA, therefore allowing to clearly identify these samples to be negative for the toxin.

In the on-site detection platform, concentrations resulting in medium signals in buffer (3.16 ng/mL for the mAb/mAb-combination and 10 ng/mL for the claudin-4/mAb-combination) resulted in a reduction in the normalized signal intensity as expected, but still provided clearly positive signals for all samples tested. In this chip-based assay, internal negative controls represented by polyclonal mouse IgG antibodies on negative control spots were subtracted from the signals on specific spots and thus allowed to differentiate between specific and non-specific signals. In conclusion, all four assays—the antibody- and receptor-based assays performed in microtiter plates and in the pBDi platform—were able to detect CPE from spiked fecal samples. 

### 2.5. Evaluation of Established ELISAs by Testing a Comprehensive Panel of C. perfringens Supernatants

The investigations presented above were performed with recombinantly produced, highly purified CPE. To also assure that native CPE produced by *C. perfringens* can be specifically detected and that other toxins or proteins released by *C. perfringens* do not interfere with the ELISAs, we tested a blinded panel of 64 *C. perfringens* supernatants carrying or not carrying the *cpe* gene. The strains had been isolated from food poisoning outbreaks or were purchased from commercial suppliers representing toxinotypes A, B, D, E, and F according to the toxinotyping scheme from Rood et al. [7]. The presence of the *cpe* gene as well as the genes for α- (*cpa*), β- (*cpb*), ε- (*etx*), ι- (*iap*), and NetB (*netB*) toxin genes were confirmed by PCR. Subsequently, *cpe*-positive and *cpe*-negative cell-free supernatants were subjected to antibody-based (CPE562 plus biotinylated CPE1) and receptor-based (claudin-4 plus biotinylated CPE562) ELISAs to detect CPE (Table 4). 

For all strains studied, results obtained from ELISA and PCR were completely consistent, irrespective of the capture reagent used for the ELISA (mAb or claudin-4): the PCR data of all *cpe-*positive supernatants were confirmed by the two different ELISAs developed. Examining the 34 *cpe-*negative supernatants in the panel, we observed no cross-reactivity of our assays towards other components present in the supernatants. In conclusion, both the receptor-based and antibody-based ELISA specifically detected native CPE present in *C. perfringens* strains isolated from different outbreaks in Germany. 

## 3. Discussion

With 5 million estimated cases per year in Europe [59], *C. perfringens* type F food poisoning is a widespread gastrointestinal disease. The causative agent of this type of food poisoning, the pore-forming toxin CPE, causes symptoms ranging from mild to fatal clinical presentations [25,60]. Furthermore, CPE is involved in up to 15% of antibiotic-associated diarrhea which is frequently connected with a high recurrence rate and long treatment durations [21]. To successfully handle these severe CPE-induced diseases, reliable fast detection systems are useful tools supporting clinicians to identify CPE as the causative agent and to provide adequate treatment in the early stages of disease. To this aim, we established classical sandwich ELISAs comprised of novel capture and detection mAbs and an innovative sandwich ELISA deploying the endogenous receptor claudin-4 as the capture reagent. After comprehensively investigating and comparing these two detection approaches in a microtiter plate format, we implemented them into the automated on-site detection platform pBDi. Both the antibody- and the receptor-based approach achieved highly sensitive detection of CPE with detection limits in the low pg/mL- (for stationary detection) and ng/mL-range (for mobile detection). Specifically, both approaches were able to detect CPE from spiked fecal samples. Furthermore, the mAb- and receptor-based microtiter plate assays correctly detected the native toxin derived from supernatants from 30 *cpe*-positive strains out of a panel of 64 *C. perfringens* strains, fully corroborating PCR results. Our results demonstrate that even complex transmembrane proteins can be functionalized as capture reagents in stationary and mobile ELISA, enabling toxin detection as sensitive and specific as classical sandwich ELISA based on mAbs only.

Surprisingly, claudin-4 was applicable as capture reagent in a microplate format and in the on-site system pBDi, although it is a 4-transmembrane protein being highly hydrophobic in nature [61]. As shown by Ling et al., the addition of a GST-tag significantly increased the solubility of a similar claudin-4 construct consisting of the extracellular and four transmembrane domains [62]. Furthermore, the same authors demonstrated the importance of the transmembrane domains for functional conformation of the claudin-4 protein [62]. Based on these findings, we observed a high affinity interaction of isolated claudin-4 towards CPE with a 24-fold lower *K_D_* (0.38 nM) compared to the reported interaction of CPE with claudin-4 expressed in intact L cells (9 nM; [63]). The high binding affinity to its target toxin suggests a native-like conformation of the recombinant claudin-4 used in our study. With a *K_D_* as low as 0.38 nM, recombinant claudin-4 revealed excellent binding properties corresponding to those of the newly generated high affinity mAbs presented in this work.

With respect to animal well-being, recombinant atoxic CPE D48A mutant was applied as immunogen for hybridoma generation [53]. Since the conformation of recombinant versus native toxin may differ, a crucial step in assay development was to verify the mAbs’ recognition of the native toxin. To this end, our experiments, using a panel of 64 *C. perfringens* supernatants mainly derived from food poisoning outbreaks, demonstrated that both the antibody- and claudin-4-based assays specifically bound native CPE in strains carrying the *cpe* gene. This finding was further supported by the fact that most antibodies prevented the toxic effect of recombinant CPE on Vero cells in vitro and, consequently, exerted neutralizing activity.

In this context, Miyamoto et al. described *C. perfringens* type E strains producing a CPE variant deviating by 10 amino acids from the classical CPE, although the consequences of this variation with respect to toxicity or illness remain unknown so far [15]. Our panel of *C. perfringens* supernatants comprised four *C. perfringens* type E strains, albeit none carried the *cpe* gene. Thus, it remains to be seen in future experiments if this variant would be successfully detected by the receptor- and the antibody-based ELISA developed in this work. Predictions about the ability of our mAbs to bind the CPE variant require further investigation on the precise epitope recognition at the amino acid level. As the receptor binding domain of the mentioned CPE variant comprises only one amino acid exchange, namely S313A, which does not contribute to receptor binding [15,38,64], we assume the interaction of claudin-4 with this variant to be as strong as with the prevalent CPE. Based on CPE’s low structural diversity described so far, the rare occurrence of type E strains in outbreak events [65,66], and our results demonstrating 100% congruence in detecting CPE from *cpe*-positive *C. perfringens* strains isolated mainly from outbreaks in Germany, as well as the potential to detect CPE from feces extracts, we strongly believe that our assays provide reliable detection of clinically relevant samples.

Along this line, due to the lack of sequence information, we currently cannot assess the potential presence of molecular variants among the CPEs produced by the 30 *cpe* positive type D or type F strains investigated in this work. Whole genome sequencing could be the subject of future work shedding light on the presence of the variant described by Miyamoto and colleagues [15] or the existence of further hitherto undescribed variants. In the case that further CPE variants exist and, analogous to other structurally diverse toxins such as shiga toxins [67], exhibit a high degree of conservation in receptor binding to retain endogenous biological activity, it is expected that the receptor-based ELISA employing claudin-4 would provide a high degree of diagnostic reliability.

To the best of our knowledge, only few classical and no receptor-based sandwich ELISA have been described for the detection of CPE until now. While some of these assays featured detection limits in the ng/mL-range [49,68], the ELISAs developed in this work achieved superior detection limits of 0.3 to 1 pg/mL depending on the capture reagent used. This is well in the range reported for a more time-consuming immuno-PCR-based ELISA (detection limit 1 pg/mL; [69]). Remarkably, the detection limit of 40 pg/mL reported for a commercial ELISA kit [70] turned out to be higher by a factor of 140 and 40 in comparison to our antibody-based and receptor-based ELISA, respectively. Contrary to previously published ELISAs, we successfully verified the performance of our microtiter plate-based ELISA using a comprehensive panel of bacterial supernatants derived from food outbreak isolates. Furthermore, the detection of CPE from spiked feces extracts was proven to be possible in the low pg/mL-range with a recovery rate of about 50% for CPE.

As with stationary detection, no receptor-based assays and only few antibody-based assays designed for rapid mobile detection of CPE have been described [48,51]. As far as sensitivity was reported, these tests were at least by a factor of 100 less sensitive compared to the LODs of 0.316 ng/mL and 1 ng/mL determined for the antibody-based and the receptor-based detection of CPE in the pBDi system, respectively. Reported concentrations of CPE in the feces of diseased patients during outbreak events were frequently in the range of 10 ng to 100 µg/g or above and only rarely between 1 to 10 ng/g in fecal samples [71,72]. Considering these reports, the pBDi’s sensitivity can be assumed to be sufficient for the successful detection of CPE from spiked clinical samples: concentrations of 3.16 ng/mL were clearly detectable in both the receptor- and antibody-based approach. For the antibody-based approach, normalized signal intensities for this concentration were in medium range of the pBDi, suggesting a potential detection limit down to 2- to 3-digit pg/mL, being well below the mentioned concentration range for real samples.

An asset of the automated pBDi platform is its capability for multiplex detection. The immobilization of distinct toxin-specific antibodies or corresponding endogenous receptors onto the gold surface of the electrodes enables the simultaneous detection of multiple toxins within one measurement. One possible approach would be to simultaneously determine the presence of CPE and the *C. difficile* toxins as a major cause for AAD in one sample, thereby allowing differential diagnosis of AAD. Prior to the application of the on-site detection assays developed for the analysis of clinical samples, the impact of different sample matrices on assay performance has to be considered. Due to their high specificity and affinity, monoclonal antibodies are usually compatible with a wide range of different matrices. In this study, the detection of CPE from spiked fecal samples, representing the most important and challenging matrix in the diagnostics of gastrointestinal diseases, has not only been shown for the classical antibody-based assay, but also for the use of claudin-4 as an innovative capture reagent in both stationary and on-site assay formats. 

While the stationary ELISA provided slightly higher but still similar recovery rates for CPE in fecal samples when using the claudin-4/mAb-detection in comparison to the mAb/mAb-detection at the tested concentrations, the fast and on-site pBDi platform delivered overall better results regarding signal intensities using the classical mAb/mAb detection approach. Although the sensitivity of the classical mAb-based detection is higher by factor three for both the stationary and the mobile detection, the receptor-based approach provides the additional opportunity to be applied as a functional assay for the receptor-binding of CPE.

Since complex matrices may give rise to non-specific signals in immunoassays leading to potential false-positive results, appropriate controls have to be applied. For real samples, empty matrices identical to the samples, that is without target toxin, are usually not available. Therefore, in case of sandwich ELISAs, this should be controlled by exchanging the specific capture reagent (antibody or receptor) by irrelevant capture reagents and to measure the complex samples in parallel in the specific and the control ELISA to identify background signals. For fecal samples tested in this study, background signals were low for the claudin-4- and the antibody-based assays and could clearly be differentiated from specific signals arising in toxin-spiked feces employing the above-mentioned controls. 

Furthermore, the presence of other proteins in complex samples might interfere with the assays by non-specific binding to the antibodies or claudin-4. For the latter one, especially endogenous tight junction proteins are known to bind to the receptor or could interact with CPE itself due to their intrinsic function. Nevertheless, due to the excellent affinities of the antibodies as well as the receptor claudin-4 to CPE, the combined specificity of the capture and detection reagents and the presentation of an excess of the detection reagents, it is considered unlikely that other proteins or matrix compounds would significantly interfere with the assays, unnoticed by the above-mentioned controls. In an unknown real sample, spiking of a defined concentration of CPE and comparison of the same concentration spiked into buffer would identify deleterious masking effects. It is noteworthy to mention that CPE from canine diarrheal samples was shown to be stable when stored for ten days and quantified by ELISA [73], thus indicating that ELISA would be able to confirm presence of CPE even from samples not measured immediately. Therefore, the herein presented assays are considered to be of high diagnostic value for the identification of CPE in real samples from diarrheal illnesses.

Apart from the demonstrated applicability in diagnostic settings, the murine mAbs generated in this work revealed excellent neutralizing properties. It therefore seems to be attractive to explore their potential use for treating AAD after humanization using recombinant techniques. Given the similar pathogenesis of AAD triggered by *Clostridium perfringens* and *Clostridioides difficile*, this could be a promising application since an already approved monoclonal antibody, bezlotoxumab, directed against *C. difficile* toxin B (TcdB) significantly reduced the recurrence of AAD induced by *C. difficile* infections [74].

## 4. Conclusions

In summary, we explored stationary and mobile ELISA-based strategies to specifically detect CPE from *C. perfringens*, either by novel high affinity mAbs detecting different epitopes or, alternatively, by binding to the endogenous receptor claudin-4 plus antibody detection. Both approaches provided comparable and excellent detection limits in the low pg/mL-range and were instrumental in successfully detecting CPE from feces extracts as well as in detecting native CPE from a panel of supernatants from clinically relevant *C. perfringens* strains isolated during food poisoning outbreaks in Germany. For the first time, we present a fast on-site detection platform identifying CPE in concentrations as low as a few ng/mL, an advancement expected to be useful in clinical laboratories to diagnose diarrhea of assumed bacterial origin. 

## 5. Materials and Methods 

### 5.1. Animal Experiments

The handling of laboratory animals complied with the regulations of the German Animal Welfare Act and European legislation for the protection of animals used for scientific purposes (Directive 2010/63/EU). Immunizations of mice to generate monoclonal antibodies (mAbs) CPE1, CPE58, CPE281, CPE384, CPE562, CPE639, and CPE1339 were approved by the State Office for Health and Social Affairs in Berlin (LAGeSo Berlin, Germany) under the registration number H129/19 (approval date 03 July 2019). The sacrifice of mice for the removal of thymocytes has been registered by the LAGeSo under the number T0060/08. With respect to animal well-being and the production of mAbs using hybridoma technology, we considered the LD_50_ of CPE (160 µg/kg body weight, systemic application; [75]) and used a recombinantly produced atoxic CPE D48A mutant for immunization which, despite having normal ability to bind and form the small complex, failed to form large complexes or induce cytotoxicity [53].

For the generation of mAbs CPE9 and CPE18, all experiments were performed in compliance with French and European regulations on the care and protection of laboratory animals (European Community (EC) Directive 2010/63/UE, French law 2001-486, 6 March 2018) and with the agreements of the Ethics Committee of the Commissariat à l’Energie Atomique (CEtEA “Comité d’Ethique en Expérimentation Animale” no. 44) no.12-026 and 15-046, delivered to S. Simon by the French Veterinary Services and CEA agreement D-91-272-106 from the Veterinary Inspection Department of Essonne (France).

### 5.2. Recombinant Expression of CPE Proteins and GST-Claudin-4 Fusion Protein

The detailed production of CPE 26–319 wild-type, the atoxic mutant CPE D48A, CPE RBD 203–319 and CPE pore forming domain (PFD) 26–202 is described elsewhere [55]. In brief, CPE 1–319 wild-type and mutant were recombinantly produced under biosafety level 2 containment (Project Number GAA A/Z 40654/3/123/7) in *Escherichia coli* B strains. Employing the *C*-terminal His6tag, proteins were purified by immobilized metal affinity chromatography (IMAC) using Co^2+^-Talon matrix (Takara Bio Europe S.A.S., Saint-Germain-en-Laye, France) and eluted with 50 mM Tris-HCl, pH 8.0, 150 mM NaCl, 250 mM imidazole. For proteolytic activation at the N-terminus and tag removal, CPE 1–319 was incubated for 16 h at room temperature with 0.08 U trypsin (Sigma–Aldrich Chemie GmbH, Steinheim, Germany) per mg CPE, yielding quantitatively tag-free CPE 26–319. Subsequent gel filtration (Superdex-75 16/60 column, Cytiva, formerly GE Healthcare, Freiburg, Germany) was performed in PBS, pH 7.4. Highly purified proteins were shock frozen in liquid nitrogen and kept at −70 °C. CPE 1–319 and CPE 26–319 were initially compared for their binding properties to antibodies and to recombinant claudin-4. Since no differences were identified, both were equally applicable as antigens in immunological assays. For the cytotoxicity assay, trypsin-activated CPE 26–319 was applied.

The detailed production of GST human claudin-4 fusion protein (GST-ShCLDN4H8) is described elsewhere [55]. In brief, GST-ShCLDN4H8 expressed in *E. coli* strain BL21 DE3 was purified in Tris/NaCl-buffer (20 mM Tris-HCl, 150 mM NaCl, pH 7.2) supplemented with 0.5% Triton X-100 (alternatively PBS containing 0.5% CHAPS) employing glutathione–sepharose beads and eluted by glutathione. Fractions containing the desired proteins were pooled and dialyzed to remove glutathione. Purified GST-ShCLDN4H8 was shock frozen in liquid nitrogen and stored at −70 °C.

### 5.3. Generation of Monoclonal Antibodies

To produce monoclonal antibodies, hybridoma technology was applied as described previously [57,76]. For the generation of mAbs CPE1, CPE58, CPE281, CPE384, CPE562, CPE639, and CPE1339, mice (BALB/c or NMRI) bred under specific-pathogen-free conditions at Charles River (Sulzfeld, Germany) were immunized at an age of about eight weeks. Immunizations were performed at least three times with an interval of four weeks. CPE RBD (5 µg) or CPE D48A (10 µg) emulsified in complete (first immunization) or incomplete Freund’s adjuvant (subsequent immunizations) were used as antigens. After achieving a high antibody titer, antigens (2 µg for CPE RBD and 6 µg for CPE D48A) diluted in phosphate-buffered saline (PBS) were injected on days −3, −2, and −1 before fusion. Spleen cells from immunized mice were fused with myeloma cells (P3-X63-Ag8.653, American Type Culture Collection) at a ratio of 2:1 in polyethylene glycol 1500 (PEG, Roche Diagnostics, Mannheim, Germany) to obtain hybridoma clones.

For the generation of mAbs CPE9 and CPE18, *Biozzi* mice were immunized 4-times at 3-week-intervals with 40 µg of detoxified CPE (detoxification of recombinant CPE provided by Michel Popoff, Institut Pasteur, Paris, France, by extensive dialysis in 0.4% formalin). The mice showing the best immune response were selected for the preparation of monoclonal antibodies and given a daily i.v. booster injection of 40 µg detoxified CPE for three days. Two days after the last boost, hybridomas were produced by fusing spleen cells with NS1 myeloma cells.

Starting on day 10, the hybridoma clones were analyzed for reactivity towards the antigens. To this end, hybridoma supernatants were tested by indirect ELISA (see below), receptor-based sandwich ELISA (using claudin-4 as capture reagent instead of capture mAb, as described below) and SPR-based methods (see below). Cell clones producing reactive supernatants were subcloned at least twice and tested by intracellular staining with Cy5-labeled anti-mouse IgG antibodies (Dianova, Hamburg, Germany) followed by flow cytometry measurement to ensure clonality. IgG-antibodies from hybridoma supernatants were purified by affinity chromatography using a HiTrap MabSelect SuRe column on an Äkta Protein Purification System (Cytiva, formerly GE Healthcare, Freiburg, Germany). The purity of mAbs was assessed by separation on SDS-PAGE under reducing conditions. To determine the isotypes of purified mAbs, a commercially available isotyping kit (Roche Applied Science, Mannheim, Germany) was used. For use as detection reagents in sandwich ELISA and pBDi platform, the purified antibodies were biotinylated using Biotin N-hydroxysuccinimide ester (Sigma Aldrich, Munich, Germany) at a molar ratio of 20 according to the manufacturer’s instructions. Biotinylated antibodies were stored in PBS containing 0.2% (w/v) bovine serum albumin (BSA; Serva, Heidelberg, Germany) and 0.05% (w/v) NaN_3_ (Carl Roth, Karlsruhe, Germany).

### 5.4. Indirect ELISA

To identify hybridomas producing antibodies specifically recognizing full length CPE (aa 1–319) and to differentiate these antibodies by the detection of the CPE domains (CPE RBD or CPE PFD), the hybridoma supernatants were analyzed by indirect ELISA. For this purpose, the cavities of MaxiSorp microtitre plates (F96; Nunc, Thermo Fisher Scientific, Langenselbold, Germany) were coated overnight at 4 °C with antigen (500 ng/mL, 50 µL per well) in PBS containing 1 µg/mL of BSA. After the plates were washed four times with 300 µL of PBS supplemented with 0.1% Tween 20 (PBS-T), blocking was performed with 200 µL of 2% skimmed milk powder (Merck, Darmstadt, Germany) in PBS-T per well for 1 h at room temperature. Plates were washed again and 50 µL of hybridoma supernatants were added and incubated for 1 h. After washing the plates, detection was achieved by incubation with 50 µL per well of horseradish peroxidase (HRP)-labeled goat-anti-mouse IgG (Fcγ) specific antibody (1:2500; Dianova, Hamburg, Germany) for 30 min. A final washing step (8×) was followed by development with 100 µL per well 3,3′,5,5′-tetramethylbenzidine (TMB, SeramunBlau slow; Seramun, Heidesee, Germany) for 15 min. The reaction was stopped by adding 100 µL of 0.25 M H_2_SO_4_ per well. Absorbance at 420 nm referenced to 620 nm was measured by an ELISA reader (Tecan; Crails-heim, Germany).

### 5.5. Antibody- and Receptor-Based Sandwich ELISA

Sandwich ELISAs were composed of a capture antibody (antibody-based) or the endogenous receptor claudin-4 (receptor-based), both combined with a detection antibody, and were performed with slight modifications as described before [77]. Briefly, capture antibodies (10 µg/mL, 50 µL per well) in PBS or claudin-4 (2.5 µg/mL, 50 µL per well) in PBS containing 1 µg/mL of BSA were immobilized onto the surface of MaxiSorp microtitre plates over night at 4 °C. Plates were washed (4×) with 300 µL of PBS-T per well. After blocking with 200 µL per well casein buffer (Diavita, Heidelberg, Germany) for 1 h and washing with PBS-T, 50 µL of antigen diluted in PBS supplemented with 0.1% BSA were incubated for 2 h at room temperature. Plates were washed again and 50 μL of hybridoma supernatant (for hybridoma screening) or biotinylated purified detection antibodies diluted in casein buffer were added and incubated for 1 h. After washing once again, 50 μL of HRP-labeled goat-anti-mouse IgG (Fcγ) specific antibody (hybridoma screening) or streptavidin-labeled poly HRP (Senova, Weimar, Germany) were incubated for 30 min. Final washing, developing and measuring were performed as described for indirect ELISA (see above).

### 5.6. Surface Plasmon Resonance (SPR) Measurements: Binding Kinetics

All SPR measurements were performed on a T200 device (Cytiva, formerly GE Healthcare, Freiburg, Germany) using sensor chips CM5 and HBS-EP+ (10 mM HEPES, pH 7.4, 150 mM NaCl, 3 mM EDTA, 0.05% Tween 20) as running buffer at 25 °C as described previously [78].

To determine binding kinetics and affinities, GST-tagged claudin-4 or monoclonal antibodies (mAbs, purified or from hybridoma supernatants) were immobilized with densities of 300 to 500 resonance units (RUs) onto the sample flow cells of a sensor chip using a GST capture kit or a mouse antibody capture kit (Cytiva, formerly GE Healthcare, Freiburg, Germany), respectively. Flow cells immobilized with recombinant GST (for claudin-4) or no ligand (for mAbs) were set as reference controls. Subsequently, the analyte was injected into all flow cells. For a fast measurement and thus a high sample throughput during hybridoma screening, SPR assays in single-cycle format were performed, successively injecting CPE at concentrations of 30.9 and 309 nM. For precise measurements (claudin-4 and purified mAbs), serial 1:3 dilutions of CPE ranging from 93 to 0.38 nM were injected sequentially in separately performed runs (multi-cycle kinetic) for 120 s at a flow rate of 30 μL/min. The ligand dissociation was initiated by injecting running buffer and was monitored for 300 s (hybridoma screening) or 1200 s (all other measurements). The sensor chip surface was regenerated using 10 mM glycine, pH 2.1 (GST capture) or 10 mM glycine, pH 1.7 (mouse antibody capture) for 120 s at a flow rate of 10 μL/min. Repro-ducibility of the measurements was controlled by duplicate injection of the highest analyte concentration at the beginning and end of the measurement cycles. All binding curves were double referenced as described [79] and fit to 1:1 Langmuir interaction models using the BIAevaluation software (3.0).

### 5.7. SPR Measurements: Epitope Binning

Epitope binning was performed to identify monoclonal antibodies with distinct epitope recognition and thus suitable sandwich combinations of two monoclonal antibodies. To this aim, the first antibody (10 µg/mL for purified mAbs or undiluted hybridoma supernatant) was immobilized via mouse antibody capture kit (Cytiva, formerly GE Healthcare, Freiburg, Germany) as capture reagent on the surface of the sample flow cell of a sensor chip at a flow rate of 10 µL/min with a contact time of 60 s. After blocking of unspecific binding on all flow cells including a control flow cell by mouse IgG (Jackson ImmunoResearch Europe, Ely, UK) (100 µg/mL, flow rate: 10 µL/min, contact time: 120 s) and injecting recombinant CPE (aa 1–319, wild type) at a concentration of 61.8 nM (all flow cells, flow rate: 10 µL/min, contact time: 60 s), the second mAb (10 µg/mL for purified mAbs or undiluted hybridoma supernatant) was injected (all flow cells, flow rate: 10 µL/min, contact time: 60 s). All binding curves were referenced to the control flow cell without CPE-specific capture antibody. The course of the curve reveals whether the two antibodies recognize the same epitope (no signal increase after injection of 2nd mAb) or distinct epitopes (signal increase detectable after injection of 2^nd^ mAb).

### 5.8. SDS-PAGE and Western Blots

Western blot analysis was used to evaluate the specificity of mAbs towards recombinant CPE (aa 26–319, wild type) and CPE domains PFD (aa 26-202; GST-PFD-t-mCherry) and RBD (aa 203-319). For SDS-PAGE, the antigens were mixed with 3 × Laemmli loading buffer (150 mM Tris-HCl, pH 6.8, 6% SDS, 30% (v/v) glycerol, 7.5% (v/v) β-mercaptoethanol, 0.25% (w/v) bromophenol blue) and heated for 10 min at 95 °C. After cooling on ice, samples were loaded onto 12% polyacrylamide gels and separated electrophoretically according to standard procedures [80]. Subsequently, gels were transferred to methanol activated Immobilon® P 0.45 μm PVDF membranes (Millipore, Schwalbach, Germany). Membranes were blocked at 4 °C overnight in 2% skimmed milk in PBS-T and incubated for 1 h at room temperature with 1 μg/mL of CPE-specific antibodies diluted in blocking buffer. After three washing steps, detection was performed by biotin-labeled goat anti-mouse IgG (Dianova, Hamburg, Germany) diluted 1:10,000 in blocking buffer for 30 min at room temperature. Membranes were developed using alkaline phosphatase-conjugated streptavidin (*Avidix*™-AP, Fisher Scientific, Bremen, Germany) and CDP-Star as substrate (Perkin Elmer, Waltham, MA, USA). Development was documented on a ChemiDoc imaging system (Bio Rad Laboratories, Hercules, CA, USA).

### 5.9. Neutralizing CPE in a Cell-Based Cytotoxicity Assay

The ability of mAbs to block the cytotoxicity of CPE in vitro was investigated by a real-time cytotoxicity assay using the impedance-based system xCELLigence (OMNI Life Science GmbH and Co, Bremen, Germany) as described [81]. Briefly, after setting the baseline, a volume of 75 µL Vero cells (ATCC, Manassas, VA, USA) was seeded into an E-plate at a concentration of 12,500 cells/well. These freshly seeded cells (no attachment to the plate) were immediately mixed with 30 µL recombinant CPE (aa 26–319, wild type) comprising the following final standard concentrations: 3.16 nM, 1 nM, 0.316 nM, 0.178 nM, 0.1 nM, 0.056 nM, 0.0316 nM, and 0.0178 nM. Standards were measured in duplicates to determine the half-maximal inhibitory concentration (IC_50_). Untreated cells supplemented with 30 µL of medium instead of toxin standard were used as the reference for viable cells. To study the neutralization of CPE by mAbs, Vero cells seeded as above were mixed on the same plate with 30 µL of a preincubated mixture (30 min at room temperature) containing recombinant CPE (aa 26–319, wild type; final concentration of 1 nM) and the respective mAb (final concentrations of 10 and 100 nM). Cell indices were monitored over 90 h. Calculated normalized cell indices are based on cell indices at 40 h after start of the incubation.

### 5.10. On-Site Detection Based on an Electrochemical Biochip Platform (pBDi)

For fast detection, the assays providing the highest sensitivity were implemented into the on-site detection platform pBDi (Bruker Optik GmbH, Leipzig, Germany). To this end, capture reagents were spotted onto electrochemical biochips as described [82] by Bruker Optik GmbH. Briefly, mAbs diluted in PBS buffer and claudin-4 diluted in PBS containing 0.5% CHAPS were immobilized in duplicate onto the surface of the gold electrodes at a final concentration of 500 µg/mL. To stabilize immobilized claudin-4, 0.4 mg/mL BSA was added as a co-immobilization agent. Biotinylated rabbit anti-mouse IgG (Sigma–Aldrich GmbH, Taufkirchen, Germany) was spotted as the positive control and mouse IgG (Jackson ImmunoResearch Europe, Ely, UK) as the negative control. Spotted biochips were provided by the manufacturer installed in polycarbonate cartridges. Before starting the measurement, all reagents and biochips were conditioned to room temperature. After diluting the lyophilized enzyme streptavidin-β-D-galactosidase, the substrate p-APG (both Bruker Optik GmbH, Leipzig, Germany) and the biotinylated detection mAb (10 μg/mL) in assay buffer (PBS, 0.5% (*w/v*) BSA, 0.5% (*w/v*) trehalose, 1 mM MgCl_2_, 0.025% (*v/v*) Tween 20), the prepared reagents and assay buffer were loaded into the pBDi instrument, 800 µL of toxin standard (CPE) diluted in assay buffer was applied to the sample compartment and the biochip was inserted. The pBDi platform performed the measurement fully automatically and the current generated by enzymatic substrate conversion was recorded by pBDi Control software (Bruker Optik GmbH, Leipzig, Germany). The mathematical normalization of the absolute current slope of target electrode positions to the signal of the positive control (PC) and negative control (NC) enabled the evaluation of inter-chip reproducibility. Normalized signals were calculated by pBDi Control software using the following equation:

(1) $$Normalized\;signal\;\left(\%\right)=\left(\frac{\;Slope\;\left(Target;\;absolut\right)\;–\;Slope\;\left(NC\right)}{Slope\;\left(PC\right)\;-Slope\;\left(NC\right)}\right)\;\times 100\%$$

### 5.11. Preparation of Spiked Fecal Samples

Feces extracts were individually prepared from the feces of three healthy donors by 1:5 (*w/v*) dilution in PBS pH 7.4 with 0.1% (*w/v*) BSA and cleared by centrifugation (10,000× *g*, 10 min). Informed consent was obtained from all donors and sample collection was non-invasively performed by the volunteers themselves. The feces extracts were spiked with recombinant wild-type CPE (1–319) at different concentrations, incubated at room temperature for 30 min and measured in stationary plate-based ELISAs and by the assays transferred to pBDi.

### 5.12. Preparation of Supernatants from Broth Cultures

For the preparation of bacterial cell culture supernatants *C. perfringens* isolates, bacteria from swabs or 50 µL of glycerol stocks were initially streaked onto lactose-egg yolk agar (LEA) plates. After one day, a number of colonies with typical *C. perfringens* morphology were transferred to 5 mL tryptone-peptone-glucose-yeast extract (TPGY) medium and cultivated for 5 to 7 days at 30 °C under anaerobic conditions (10% H_2_, 10% CO_2_, 80% N_2_) in an anaerobic workstation (A35 or A85, Don Whitley Scientific, Shipley, West Yorkshire, UK). The supernatants obtained after clarification (12,000× *g*, 5 min, 4 °C) were stored at −20 °C or −80 °C. For ELISA measurements, supernatants were diluted 1:10 (*v/v*) in PBS supplemented with 0.1% BSA.

### 5.13. Gene Toxinotyping by qPCR

*C. perfringens* isolates were toxinotyped based on the recently updated typing scheme by Rood et al., 2018 [7]. This typing relies on the detection of the toxin genes for α- (*cpa*), β- (*cpb*), ε- (*etx*), ι-toxin (*iap*), enterotoxin (*cpe*), and NetB toxin (*netB*). PCR for *netB* was performed as described [83]. For the detection of *cpa, cpb, etx, iap,* and *cpe* genes, two alternative quantitative PCRs were applied. For strains from the Bavarian Health and Food Safety Authority (LGL), primer and probe sequences as well as PCR conditions were as published previously [84]. For all other strains, primer and probes were newly designed with the Primer3 build-in in the Geneious software package (Biomatters Ltd., Auckland, New Zealand) and are listed in Supplementary Table S1. The final primer and probe concentrations were 250 and 150 nM, respectively. PCR was performed for 45 cycles with denaturation at 95 °C for 15 s following elongation for 1 min at 60°C (*cpa, cpb, iap*) or 62 °C (*cpe, etx*).

## Figures and Tables

**Table 1:** Overview of reagents for the detection of CPE established and investigated in this study.

Epitope ^a^	Reagent	Source	Isotype	Specificity ^b^	Neutralization ^c^	*k*_a_^d^ (M^−1^ s^−1^)	*k*_d_^d^ (s^−1^)	Affinity ^d^ *K_D_* (M)
1	mAb CPE1	RKI	IgG1	RBD	Yes	1.0 × 10^6^	5.4 × 10^−5^	5.3 × 10^−11^
1	mAb CPE9	CEA	IgG1	RBD	Yes	6.7 × 10^5^	2.6 × 10^−5^	3.9 × 10^−11^
1	mAb CPE58	RKI	IgG1	RBD	Yes	6.9 × 10^5^	1.6 × 10^−3^	2.3 × 10^−9^
1	mAb CPE281	RKI	IgG1	RBD	Yes	7.5 × 10^5^	1.0 × 10^−3^	1.4 × 10^−9^
2	mAb CPE639	RKI	IgG1	PFD	No	4.1 × 10^5^	8.6 × 10^−4^	2.1 × 10^−9^
3	mAb CPE562	RKI	IgG1	PFD	Yes	3.1 × 10^5^	6.8 × 10^−5^	2.2 × 10^−10^
3/4	mAb CPE18	CEA	IgG1	PFD	Yes	4.6 × 10^5^	2.0 × 10^−4^	4.4 × 10^−10^
4	mAb CPE384	RKI	IgG1	PFD	Yes	2.7 × 10^5^	4.9 × 10^−4^	1.9 × 10^−9^
5/4	mAb CPE1339	RKI	IgG1	RBD	Yes	1.2 × 10^6^	1.3 × 10^−5^	1.1 × 10^−11^
1	rec. claudin-4	MHH	−	RBD	n.t.	5.6 × 10^5^	2.1 × 10^−4^	3.8 × 10^−10^

^a^ Measured by surface plasmon resonance (SPR) epitope binning using recombinant *Clostridium perfringens* enterotoxin (CPE) (aa 1–319, wild type), arbitrary consecutive numeration; for binding curves see Supplementary Figure S1. ^b^ Measured by Western blotting (Supplementary Figure S2) and SPR binding analysis (Supplementary Figure S3). ^c^ Determined by a cell-based in vitro assay using the impedance-based system xCELLigence and recombinant CPE (aa 26–319, wild type) (Supplementary Figure S4). ^d^ Analyzed by SPR measurements using recombinant CPE (aa 26–319, wild type) as analyte, for binding curves see Supplementary Figure S5. RKI = Robert Koch Institute, CEA = French Alternative Energies and Atomic Energy Commission, MHH = Medizinische Hochschule Hannover, PFD = N-terminal pore-forming domain (aa 26-202; GST-PFD-t-mCherry) of CPE, RBD = receptor binding domain (aa 203–319) of CPE, rec. claudin-4 = recombinant claudin-4 (GST-ShCLDN4H8), n.t. = not tested.

**Table 2:** Sensitivities (half maximal effective concentration (EC_50_) in ng/mL) of mAb/mAb- and claudin-4/mAb combinations integrated into sandwich ELISA *.

Domain Specificity	Capture	Detection				
		CPE1	CPE639	CPE562	CPE384	CPE1339
RBD	CPE1	-	0.20	**0.04**	0.26	0.08
PFD	CPE639	0.11	-	0.61	0.22	0.13
PFD	CPE562	0.06	3.14	-	0.16	0.11
PFD	CPE384	0.15	0.28	0.11	-	1.17
RBD	CPE1339	0.10	0.32	0.08	3.47	-
RBD	Rec. claudin-4	-	0.53	**0.16**	1.81	0.63

* Sensitivities are represented by EC_50_ values in ng/mL derived from measurement curves obtained from serial dilutions of recombinant CPE (aa 1–319). Most sensitive mAb/mAb and claudin-4/mAb combinations, respectively, are indicated in bold font. RBD = receptor binding domain of CPE, PFD = N-terminal pore-forming domain of CPE. Sorting of table according to epitope groups as given in Table 1.

**Table 3:** Detection limits for the detection of CPE in sandwich ELISA and pBDi platform.

Capture	Detection	Platform	EC_50_ (pg/mL) ^a,b^	LOD (pg/mL) ^c,d^
mAb CPE1	mAb CPE562	ELISA	13.7 ± 5.9 ^a^	0.28 ± 0.16 ^c^
		pBDi	3160 ^b^	316 ^d^
rec. claudin-4	mAb CPE562	ELISA	47.2 ± 5.1 ^a^	1.03 ± 0.30 ^c^
		pBDi	10000 ^b^	1000 ^d^

^a^ The half maximal effective concentration (EC_50_) for ELISA was determined from five independent standard curves (n = 5, mean ± SD). ^b^ EC_50_ for the pBDi is the measured concentration most closely to half maximum signal intensity, measured on two biochips with two target positions each as given in Figure 2B (n = 4). ^c^ The limit of detection (LOD) for ELISA was calculated from the data in Figure 2A with mean + 3.29 × SD of 5 blank values interpolated into the standard curves (n = 5, mean ± SD). ^d^ The detection threshold for pBDi was calculated from mean + 3.29 × SD of 8 blank values. LOD is the lowest concentration that exceeded the threshold, measured on two biochips with two target positions each as given in Figure 2B (n = 4). CPE = recombinant CPE (aa 1–319, wild type); rec. claudin-4 = recombinant construct GST-ShCLDN4H8.

**Table 4:** Detection of native CPE derived from *Clostridium perfringens* supernatants.

Strain	Toxinotype ^a^	Reference	PCR *cpe*	Sandwich ELISA ^b^	
				mAb/mAb	Claudin-4/mAb
61a	F	Miprolab ^c^	+	+++	+++
11-13136	D	LGL ^d^	−	−	−
21638/07-L574	D	LGL ^d^	−	−	−
HF 2109	A	LGL ^d^	−	−	−
HF 2110-2	A	LGL ^d^	−	−	−
11-44702	D	LGL ^d^	−	−	−
10-70639	D	LGL ^d^	−	−	−
3570/08	A	LGL ^d^	−	−	−
SO2253/1	D	LGL ^d^	−	−	−
11-30379-03	D	LGL ^d^	−	−	−
KV3 29.05	D	LGL ^d^	−	−	−
12-45390	D	LGL ^d^	−	−	−
12-45681	D	LGL ^d^	+	+++	+++
SO 21002	D	LGL ^d^	−	−	−
11.-2294	E	LGL ^d^	−	−	−
BA 204	D	LGL ^d^	−	−	−
PP 42138-15	F	LGL ^d^	+	+++	+++
12-102988	D	LGL ^d^	−	−	−
SO 221	A	LGL ^d^	−	−	−
15-0273682	D	LGL ^d^	+	+++	+++
11-4999/1	D	LGL ^d^	−	−	−
11-18210	D	LGL ^d^	−	−	−
S 726	D	LGL ^d^	−	−	−
E 728	F	LGL ^d^	+	+++	+++
L 443/05	F	LGL ^d^	+	+++	+++
21439/07 - G 1144	F	LGL ^d^	+	+++	+++
6466/08	F	LGL ^d^	+	+++	+++
10-0058087-1	F	LGL ^d^	+	++	++
6682/1	F	LGL ^d^	+	+++	+++
10-0029262-001-01 L93	A	LGL ^d^	−	−	−
10-70711/4	F	LGL ^d^	+	+++	+++
11-162672	D	LGL ^d^	−	−	−
12-45390	D	LGL ^d^	−	−	−
12-105747 L362	F	LGL ^d^	+	+++	++
P V4 8.7.	E	LGL ^d^	−	−	−
A202	E	LGL ^d^	−	−	−
14-130465	F	LGL ^d^	+	+++	+++
17-52183-001	F	LGL ^d^	+	+++	+++
17-52386-001	F	LGL ^d^	+	+++	+++
12-73336_G737/1	F	LGL ^d^	+	+++	+
E730	F	LGL ^d^	+	++	+++
PS8150/07	F	LGL ^d^	+	++	+
175-8/97	F	LGL ^d^	+	+++	+++
MB30 o.H.	F	LGL ^d^	+	++	+++
11 1331	F	LGL ^d^	+	+++	+++
E732	F	LGL ^d^	+	++	+++
12-134928_L457	F	LGL ^d^	+	+	+
PS10950/07	A	LGL ^d^	−	−	−
F436	A	LGL ^d^	−	−	−
VA00249/12	D	TLV ^e^	+	+++	+++
VA00807/14	D	TLV ^e^	+	+++	+++
VA00084/19	D	TLV ^e^	+	+++	+++
572c	D	Miprolab ^c^	−	−	−
NCTC 8239-01	F	NCTC ^f^	+	+++	++
NCTC 8798-01	F	NCTC ^f^	+	+++	+++
NCTC8346	D	NCTC ^f^	−	−	−
NCTC13110	B	NCTC ^f^	−	−	−
NCTC3110	B	NCTC ^f^	−	−	−
NCTC6121	B	NCTC ^f^	−	−	−
NCTC8084	E	NCTC ^f^	−	−	−
NCTC8238	F	NCTC ^f^	+	++	+
NCTC8504	D	NCTC ^f^	−	−	−
NCTC8533	B	NCTC ^f^	−	−	−
NCTC9851	F	NCTC ^f^	+	+++	+++

^a^ Presence of genes *cpa, cpb, etx, iap, cpe,* and *netB* was determined by qPCR and toxinotypes are given according to Rood et al. [7]. ^b^ Absorbance A in sandwich ELISA measured at wavelength 450 nm subtracted by the absorbance at reference wavelength 620 nm using mAbs CPE562 and biotinylated CPE1 for “mAb/mAb” and claudin-4 plus biotinylated CPE562 for “Claudin-4/mAb”: “−” A < 0.2; “+” 0.2 ≤ A ≤ 1.0; “++” 1.0 ≤ A ≤ 2.0; “+++” A > 2.0. ^c^ Frank Gessler, miprolab GmbH, Göttingen, Germany. ^d^ Ute Messelhäußer, Bavarian Health and Food Safety Authority (LGL), Oberschleißheim, Germany. ^e^ Dagmar Rimek, Thuringian State Authority for Consumer Protection (TLV), Bad Langensalza, Germany. ^f^ PHE Culture Collections, Salisbury, United Kingdom.

## Data Availability

Data is contained within the manuscript or the supplementary information. The data presented in this study is available in Neumann, T.; Krüger, M.; Weisemann, J.; Mahrhold, S.; Stern, D.; Dorner, M.B.; Feraudet-Tarisse, C.; Pöhlmann, C.; Schulz, K.; Messelhäußer, U.; Rimek, D.; Gessler, F.; Elßner, T.; Simon, S.; Rummel, A. and Dorner, B.G. Innovative and Highly Sensitive Detection of *Clostridium perfringens* Enterotoxin Based on Receptor Interaction and Monoclonal Antibodies.

## Supplementary Materials

The following are available online at https://www.mdpi.com/article/10.3390/toxins13040266/s1, Figure S1. Characterization of mAbs deploying SPR-based epitope binning. Figure S2. Specificity of anti-CPE mAbs for toxin subdomains by Western blotting. Figure S3. SPR measurements demonstrating domain specificity. Figure S4. Neutralization of CPE by mAbs in a cell-based in vitro assay using the impedance-based system xCELLigence. Figure S5. SPR measurements revealing kinetic binding properties of mAbs generated in this work. Table S1. Primers and probes designed in this work to detect *C. perfringens’* toxin genes.
