# Supplementary material for: Innovative and Highly Sensitive Detection of Clostridium perfringens Enterotoxin Based on Receptor Interaction and Monoclonal Antibodies
Source: Toxins (Basel). 2021 Apr 8;13(4):266. doi: 10.3390/toxins13040266 (PMC8068247; doi:10.3390/toxins13040266)
Supplement: Supplementary file 1 [file toxins-13-00266-s001.pdf]

Article

Supplementary Materials:Innovative and highly sensitive detection of *Clostridium perfringens* enterotoxin based on receptor interaction and monoclonal antibodies

Thea Neumann, Maren Krüger, Jasmin Weisemann, Stefan Mahrhold, Daniel Stern, Martin B. Dorner, Cécile Feraudet-Tarisse, Christopher Pöhlmann, Katharina Schulz, Ute Messelhäußer, Dagmar Rimek, Frank Gessler, Thomas Elßner, Stéphanie Simon, Andreas Rummel and Brigitte G. Dorner

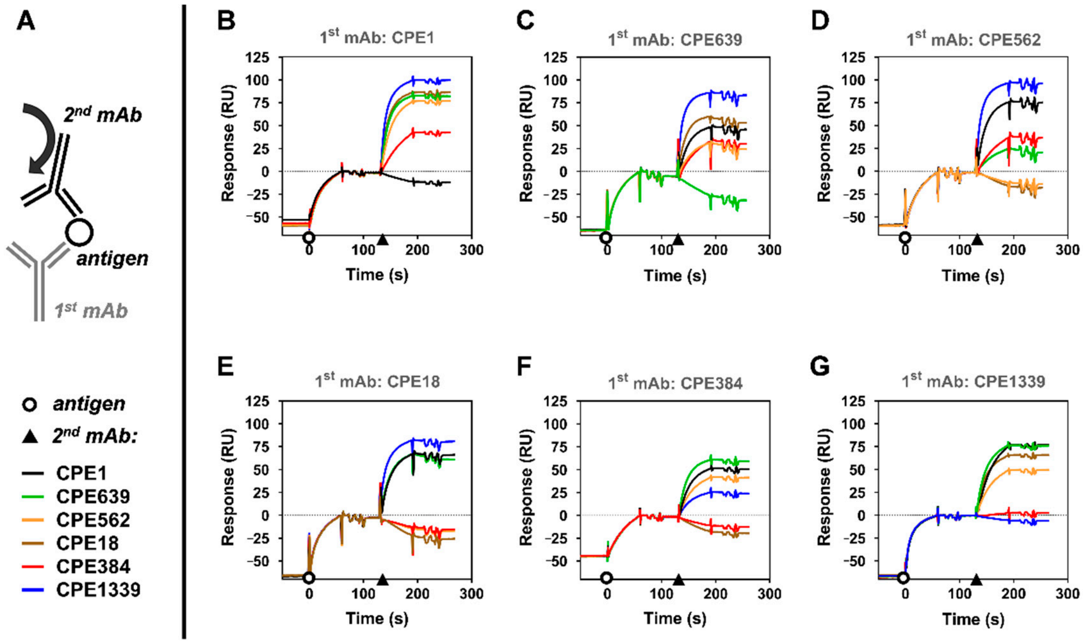

Figure S1. Characterization of mAbs deploying SPR-based epitope binning. **A.** The assay setup on the chip corresponds to a sandwich ELISA. The capture antibody (1st mAb) was immobilized via a mouse antibody capture kit on the surface of a CM5 sensor chip. After blocking of nonspecific binding sites by nonspecific polyclonal mouse mAbs and injecting recombinant CPE (aa 1–319, wild type; 2 µg/mL, open circle), the second monoclonal antibody (2nd mAb, black triangle) was injected. The curve progression reveals whether two antibodies recognize the same epitope (no signal increase after injection of 2nd mAb) or distinct epitopes (signal increase after injection of 2nd mAb). Curves of one representative binning experiment are shown. **B.** Epitope 1, recognized by mAb CPE1. Since mAbs CPE9, CPE58 and CPE281 revealed same epitope recognition (and therefore similar curves), instead of showing all data only representative curves of mAb CPE1 are shown. **C.** Epitope 2 recognized by mAb CPE639. **D.** Epitope 3, recognized by mAb CPE562. **E.** mAb CPE18 recognizing an epitope overlapping with epitopes 3 and 4. **F.** Epitope 4, recognized by mAb CPE384. **G.** Epitope 5, recognized by mAb CPE1339, overlapped with epitope 4. Sorting of antibodies in the Figure according to epitope groups indicated in Table 1 of the main manuscript.

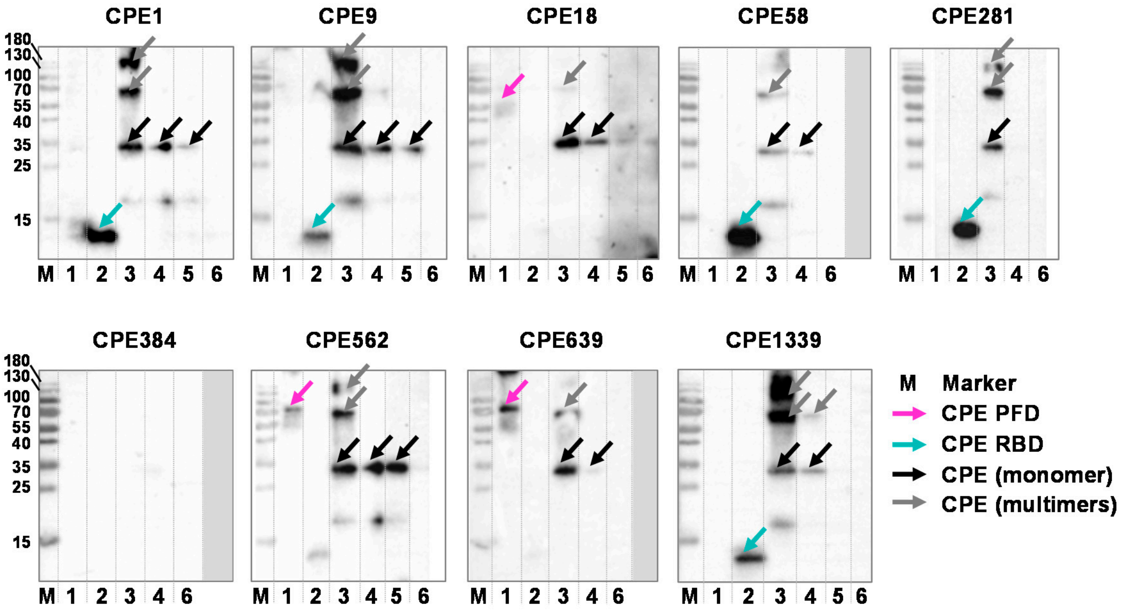

Figure S2. Specificity of anti-CPE mAbs for toxin subdomains by Western blotting. Recombinantly expressed CPE PFD (aa 26–202, GST-PFD-t-mCherry; 228 ng in lane 1), CPE RBD (aa 203–319; 41 ng in lane 2), CPE full length (aa 26–319, wild type; 100 ng in lane 3, 10 ng in lane 4 and 1 ng in lane 5) and ε-toxin (negative control, lane 6) were separated by SDS-PAGE (12%) and transferred onto PVDF membranes by electroblotting. The membranes were incubated with the indicated antibodies (clone names above each panel) followed by detection with biotinylated anti-mouse antibody and streptavidin-alkaline phosphatase-conjugate and CDP-Star as chemiluminescent substrate. Specificity of detected bands is labeled by colored arrows.

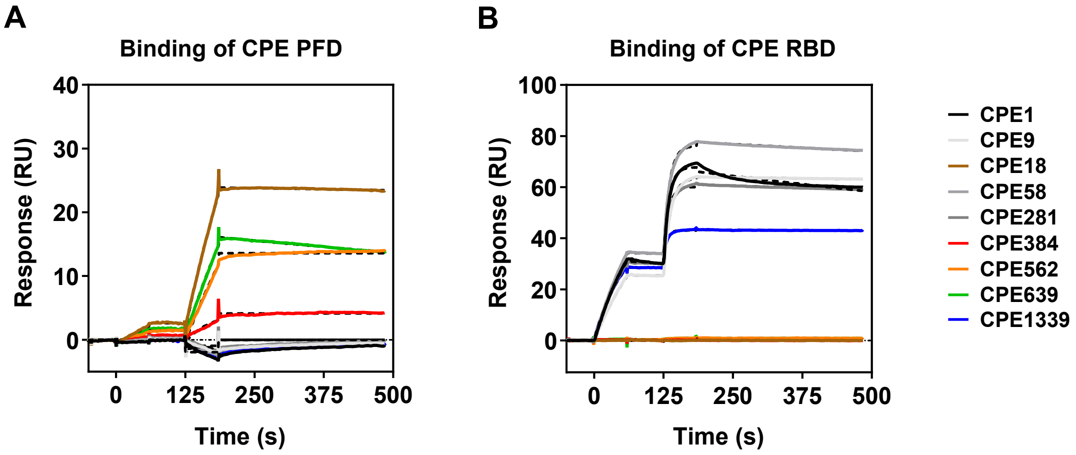

Figure S3. SPR measurements demonstrating domain specificity. Monoclonal antibodies were characterized by SPR analysis using recombinant CPE PFD (aa 26–202, GST-PFD-t-mCherry) (A) and recombinant CPE RBD (aa 203–319) (B) as antigens. After immobilizing antibodies via a mouse antibody capture kit (GE Healthcare) onto the chip surface, two concentrations of the antigens (200 and 2000 nM for CPE PFD as well as 40 and 400 nM for CPE RBD) were injected in single-cycle modus. One representative experiment out of two (CPE PFD) or one (CPE RBD) is shown.

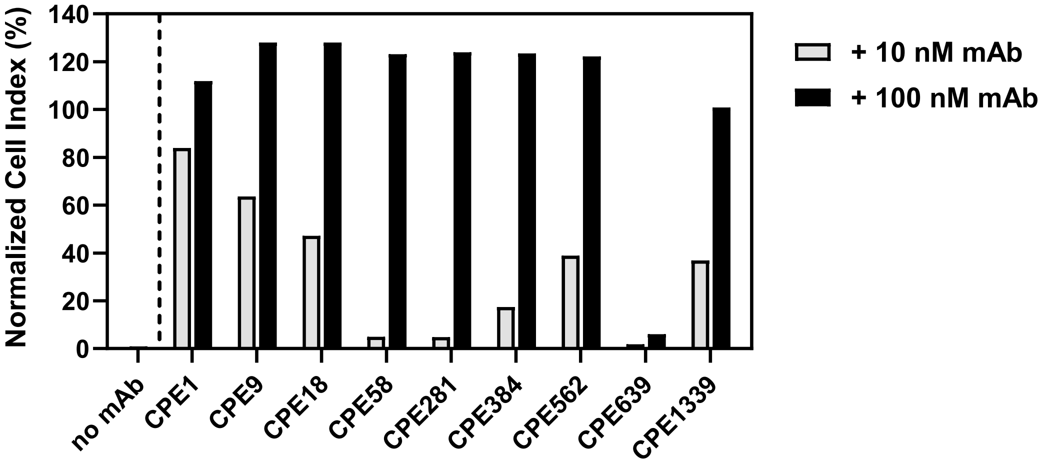

Figure S4. Neutralization of CPE by mAbs in a cell-based *in vitro* assay using the impedance-based system xCELLigence. After setting the baseline, Vero cells (ATCC, Manassas, VA, USA) were seeded into an E-plate. Subsequently, freshly seeded cells were incubated for 40 h with a preincubated mixture containing recombinant CPE (aa 26–319, wild type; final concentration of 1 nM) and the respective mAb (final concentrations 10 or 100 nM). Untreated cells (no CPE and no mAb) were carried along in separate wells as live control resulting in high cell indices (corresponds to normalized cell index of 100%). Cell indices measured for the samples were referenced to the live control yielding the normalized cell indices. Low normalized cell indices reveal a weak protection against CPE cytotoxicity and therefore a high number of dead cells. In contrast, mAbs achieving high normalized cell indices led to a high number of surviving cells. Results derived from one representative experiment (n=1) are shown.

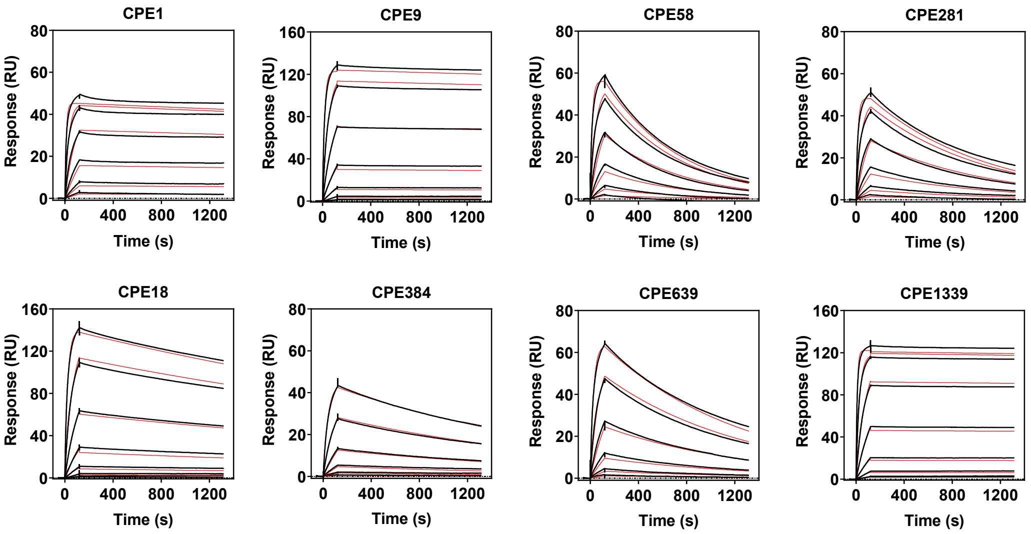

Figure S5. SPR measurements revealing kinetic binding properties of mAbs generated in this work. Antibodies (clone names above each panel) were immobilized onto a sensor chip using a mouse antibody capture kit. Serial 1:3 dilutions of recombinant CPE (aa 26–319, wild type; starting with 90 nM) were injected in separately performed runs (multi-cycle kinetic). Data were fitted using the 1:1 Langmuir binding model (black lines = measured sensorgram; red lines = fit). See Table 1 for results of these measurements and calculated kinetic data. Sensorgrams for mAb CPE562 are given in Figure 1D in the main manuscript.

Table S1. Primers and probes designed in this work to detect *C. perfringens'* toxin genes.

Primer or probe[a]	Sequence (5′ → 3′)	Amplicon size
cpa-F2<br>cpa-R3<br>cpa-P2	CTAACTCTCAAAAAGGAACAGCAGG<br>ACATGTAGTCATCTGTTCCAGCATC<br>[ATTO647]ACACGATGTATCAGAGGGTAATGATCCATCAGTTGG[BBQ650][b]	197 nt
cpb1-F2<br>cpb1-R2<br>cpb1-P2	AAAGCTTCAATTGAAAGCGAATATGC<br>TACATTTGGGGTATCAAAAGCTAGC<br>[TxRed]ACAGACAGATCATTCAACCTCTAAAGCTTCATGGGA[BHQ2]	429 nt
cpe-F2<br>cpe-R3<br>cpe-P2	TATCTACAACTGCTGGTCCAAATGA<br>GAACATATTGTCCAGCATCTAAGC<br>[FAM]ACACGATGTATCAGAGGGTAATGATCCATCAGTTGG[BHQ1]	540 nt
etx-F2<br>etx-R2<br>etx-P2	TCTTGAAGATGTTTATGTTGGAAAAGC<br>AAGGAACAGTAAACTTAGCAGTTGC<br>[HEX]ACTGCAACTACTACTCATACTGTGGGAACTTCGA[BHQ2]	167 nt
iap-F1<br>iap-R1<br>iap-P1	ACTATATGCGTGGAGGATATACYGC<br>GAATGGCTGATAAATAAGCTCCTGG<br>[HEX]AGGTCTGGTCCWCAAGAATTTGGATTAACTCTCACA[BHQ2]	384 nt

[a]: F, forward primer; R, reverse primer; P, probe are indicated for the *cpe* gene as well as for the genes for α- (cpa), β- (cpb), ε- (etx), ι- (iap) toxin genes. [b]: Fluorescent dyes and quenchers used are indicated.
